# Insomnia Symptoms and Daytime Fatigue Co-Occurrence in Adolescent and Young Adult Childhood Cancer Patients in Follow-Up after Treatment: Prevalence and Associated Risk Factors

**DOI:** 10.3390/cancers14143316

**Published:** 2022-07-07

**Authors:** Shosha H. M. Peersmann, Martha A. Grootenhuis, Annemieke van Straten, Wim J. E. Tissing, Floor Abbink, Andrica C. H. de Vries, Jacqueline Loonen, Helena J. H. van der Pal, Gertjan J. L. Kaspers, Raphaële R. L. van Litsenburg

**Affiliations:** 1 Princess Máxima Center for Pediatric Oncology, 3584 CS Utrecht, The Netherlands; s.h.m.peersmann@prinsesmaximacentrum.nl (S.H.M.P.); m.a.grootenhuis-2@prinsesmaximacentrum.nl (M.A.G.); w.j.e.tissing@prinsesmaximacentrum.nl (W.J.E.T.); a.c.h.devries-15@prinsesmaximacentrum.nl (A.C.H.d.V.); h.j.h.vanderpal@prinsesmaximacentrum.nl (H.J.H.v.d.P.); g.j.l.kaspers@prinsesmaximacentrum.nl (G.J.L.K.); 2 Emma Children’s Hospital, Amsterdam UMC, Vrije Universiteit Amsterdam, 1081 HV Amsterdam, The Netherlands; f.abbink@amsterdamumc.nl; 3 University Medical Center Utrecht, Wilhelmina Children’s Hospital, 3584 CX Utrecht, The Netherlands; 4 Department of Clinical, Neuro, and Developmental Psychology, Faculty of Behavioural and Movement Science, Amsterdam Public Health Research Institute, Vrije Universiteit Amsterdam, 1081 HV Amsterdam, The Netherlands; a.van.straten@vu.nl; 5 Beatrix Children’s Hospital, University Medical Center Groningen, University of Groningen, 9713 GX Groningen, The Netherlands; 6 Erasmus MC—Sophia Children’s Hospital, Department of Pediatric Hemato-Oncology, 3015 GD Rotterdam, The Netherlands; 7 Department of Hematology, Radboud University Medical Center, 6525 GA Nijmegen, The Netherlands; jacqueline.loonen@radboudumc.nl

**Keywords:** childhood cancer, sleep, fatigue, adolescents and young adults (AYA), survivors, quality of life

## Abstract

**Simple Summary:**

Insomnia symptoms and daytime fatigue significantly impact physical and psychosocial health. While these are common symptoms in pediatric oncology, relationships between these symptoms remain unclear. This study evaluated the prevalence of insomnia only, daytime fatigue only, the co-occurrence of insomnia and daytime fatigue symptoms, and associated risk factors in adolescent/young adult childhood cancer patients in follow-up after treatment. Results showed that around forty percent had insomnia and daytime fatigue symptoms, which often co-occurred. Risk factors that emerged were: female sex and co-morbidities (all), shorter time after treatment and bedtime gaming (insomnia only), young adulthood (insomnia–fatigue and fatigue only), needing someone else to fall asleep and inconsistent wake times (both insomnia groups), and lower educational level and consistent bedtimes (insomnia–fatigue). Overall, insomnia symptoms and daytime fatigue were common and often co-occurred in this patient population. While current fatigue guidelines do not include insomnia symptoms, healthcare providers should inquire about insomnia as this potentially provides additional options for treatment and prevention.

**Abstract:**

Insomnia symptoms and daytime fatigue commonly occur in pediatric oncology, which significantly impact physical and psychosocial health. This study evaluated the prevalence of insomnia only, daytime fatigue only, the co-occurrence of insomnia–daytime fatigue symptoms, and associated risk factors. Childhood cancer patients (*n* = 565, 12–26 years old, ≥6 months after treatment) participated in a national, cross-sectional questionnaire study, measuring insomnia symptoms (ISI; Insomnia Severity Index) and daytime fatigue (single item). Prevalence rates of insomnia and/or daytime fatigue subgroups and ISI severity ranges were calculated. Multinomial regression models were applied to assess risk factors. Most patients reported no insomnia symptoms or daytime fatigue (61.8%). In the 38.2% of patients who had symptoms, 48.1% reported insomnia and daytime fatigue, 34.7% insomnia only, and 17.1% daytime fatigue only. Insomnia scores were higher in patients with insomnia–daytime fatigue compared to insomnia only (*p* < 0.001). Risk factors that emerged were: female sex and co-morbidities (all), shorter time after treatment and bedtime gaming (insomnia only), young adulthood (insomnia–fatigue/fatigue only), needing someone else to fall asleep and inconsistent wake times (both insomnia groups), lower educational level and consistent bedtimes (insomnia–fatigue). Insomnia symptoms and daytime fatigue are common and often co-occur. While current fatigue guidelines do not include insomnia symptoms, healthcare providers should inquire about insomnia as this potentially provides additional options for treatment and prevention.

## 1. Introduction

Sleep problems are common in adolescents and young adults after childhood cancer treatment: approximately 30% report sleep problems, which significantly impacts their health and quality of life [1,2,3]. Sleep problems can be diverse, but the most common type after childhood cancer is characterized by insomnia complaints [4]. Insomnia can be defined as difficulty initiating or maintaining sleep during the night, with impaired sleep quality, which has a substantial impact on daily functioning [5]. Daytime consequences of insomnia may include: fatigue, sleepiness, mood disturbances, and impaired work/school functioning or cognitive difficulties, e.g., memory or concentration problems [6]. However, not all patients experience similar consequences during the day [6,7,8]; for example, not all patients report comparable levels of fatigue [8,9,10].

In pediatric oncology, another commonly reported problem in survivors is fatigue; rates vary between 10 and 85% depending on how fatigue is assessed and in which population [11,12]. Survivors themselves describe fatigue as one of the most substantial side effects of cancer and treatment [13]. Sleep disturbances and fatigue in (pediatric) oncology are generally assumed to be multifactorial, including biological, psychological, and social factors [14,15,16,17,18], and where predisposing, perpetuating, and maintaining factors play a role. Fatigue and insomnia seem to be related in a bidirectional manner, with fatigue being a maintaining factor for insomnia and vice versa. This may occur through disturbances of the sleep–wake rhythm with increased napping and inconsistent sleep habits, or decreased physical activity [18,19,20,21,22].

However, much is unclear about this bidirectional relationship between insomnia and fatigue in pediatric oncology. The prevalence of co-occurring insomnia symptoms and daytime fatigue after treatment is unclear, since they are often studied separately and not assessed together [2,23,24,25]. Only two studies with small sample sizes evaluated their interrelationship: in a qualitative study of 35 fatigued childhood cancer survivors, none reported sleep difficulties [13]. However, in a study of 62 long-term survivors of childhood lymphomas and leukemia, 59% of those with persistent chronic fatigue had insomnia versus 21% in non-fatigued survivors [26]. In addition, some studies after treatment did report a correlation between sleep and fatigue [27], sleep–fatigue symptom clusters [28], or found sleep disturbance as a predictor of fatigue [29]. In adult oncology, an interrelationship between impaired sleep and fatigue in survivors using different methods and in diverse populations has been reported [30,31,32]; however, much remains unclear about the specific mechanisms of co-occurring insomnia and fatigue. Since studies in large samples in pediatric oncology are lacking, little information about sleep has been included within recommendations for the surveillance and treatment of cancer-related fatigue in pediatric oncology survivors [11,33]. More knowledge is needed about the co-occurrence of insomnia symptoms and daytime fatigue, since insomnia can be effectively treated [34] and this might contribute to relieving the burden of daytime fatigue [35,36]. Additionally, risk factors for co-occurring insomnia symptoms and daytime fatigue need to be explored to identify patients who suffer from these symptoms.

Therefore, the main aim of this study was to estimate the prevalence of insomnia symptoms and daytime fatigue and to explore the co-occurrence of these symptoms in adolescents and young adults in follow-up after childhood cancer treatment. Furthermore, we assessed risk factors of patients with (1) insomnia and daytime fatigue, (2) insomnia only, and (3) daytime fatigue only, compared to patients with no symptoms.

## 2. Materials and Methods

### 2.1. Study Participants

This study is part of the MICADO project (Managing Insomnia after Childhood cancer in ADOlescents), a two-step project aimed at screening for and treatment of sleep problems in pediatric oncology groups. The data for the current study were collected during part 1 of the study, with a cross-sectional national cohort design. For more information on the procedures and characteristics of responders and non-responders to the questionnaires, please see Peersmann et al. [4]. Adolescents and young adults in the Netherlands that were included in the Dutch Childhood Oncology Group registry (DCOG; patients that were 6 months up to 5 years after diagnosis) or the Dutch Childhood Cancer Survivorship Study (DCC-SS LATER registry; patients that were 5–10 years after diagnosis) were eligible if they: (1) were aged ≥12 years old at the time of study; (2) were diagnosed with cancer or a non-malignant/low-grade tumor for which oncologic follow-up was required, before the age of 19 years; (3) were diagnosed within the last 10 years; and (4) had finished their last cancer treatment at least 6 months prior. Exclusion criteria were: (a) receiving palliative therapy, (b) not being able to fill out questionnaires independently due to a language barrier or cognitive impairment, (c) physician-specific reasons, and (d) comorbidities that prohibited effective or safe participation in part 2 of the project, which included an intervention for insomnia: severely diminished vision with no light perception, schizophrenia, or severe substance abuse [37]. Although patients in follow-up care as well as survivors were included in this study, all participants are referred to as patients in follow-up after treatment.

### 2.2. Procedures

We recruited eligible patients between November 2018 and July 2021 from five locations: the Princess Máxima Center for pediatric oncology in Utrecht, Amsterdam University Medical Center, University Medical Center Groningen, Radboud University Medical Center Nijmegen, and Erasmus Medical Center Rotterdam. It was communicated to the invited patients that they could participate with and without sleep problems, to ensure a representative sample. To compensate for their time and effort, a gift card was sent to the participants. Informed consent was obtained from all participants and parents of children aged below 16 years. The Institutional Review Board of the University Medical Center Utrecht classified this study (18-470) as exempt from the Medical Research Involving Human Subjects Act (WMO, article 16).

### 2.3. Measures

#### 2.3.1. Insomnia Severity Index (ISI)

The ISI is a commonly used instrument to assess the severity of insomnia symptoms [38,39]. This self-report questionnaire includes 7 items, which are rated on a 5-point Likert scale assessing insomnia symptoms in the last seven days. The scores range from 0 to 28 and clinical cut-off points have been determined, including: 0–7, no clinically significant insomnia; 8–14, subthreshold insomnia; 15–21, clinical insomnia—moderate severity; and 22–28, clinical insomnia—severe [39]. All participants with a score of 8 or more were classified as having insomnia symptoms. This cut-off was used as recommended in a study with young adult cancer survivors [40]. The psychometric properties of the ISI are well established [39] and have also been investigated in young adult cancer survivors [40] and adolescents [41]. The reliability coefficient (internal consistency) in this study was good (Cronbach’s α = 0.90).

#### 2.3.2. Daytime Fatigue

Daytime fatigue was measured with one self-report item derived from the Holland Sleep Disorder Questionnaire (HSDQ) [42,43], “I feel fatigued during the day”, in the past three months. The item was rated on a Likert scale ranging from 1 to 5. Participants with a score of 4 (mostly applicable) or 5 (completely applicable) were classified as having symptoms of daytime fatigue. Participants with a lower score (1 = not all applicable, 2 = mostly not applicable, and 3 = sometimes not, sometimes applicable) were classified as not having symptoms of daytime fatigue.

#### 2.3.3. Risk Factors

Sociodemographic variables (age, sex, country of birth, educational level, living situation) were measured using an additional self-report questionnaire. We categorized age as adolescents (aged 12–17 years old) and young adults (18–26 years old) and educational level as low, middle, and high, in line with the Dutch standard education classification [44]. The self-report questionnaire also included childhood cancer-related questions about the type of cancer diagnosis, type of cancer treatment, and age at diagnosis. Furthermore, it included a question about the presence of co-morbid health conditions (open-ended question), which was dichotomized (yes/no). In Supplementary Table S1, more information is provided about the types of co-morbid health conditions that were reported by participants. The DCOG and DCC-SS-LATER databases were used to verify age at study invitation, sex, age at diagnosis, time since diagnosis, and type of cancer diagnosis for non-responders [4].

To assess sleep behaviors, we used items from the Pediatric Sleep Practices Questionnaire (PSPQ) [45]: needing someone else to fall asleep (either a parent or a significant other, such as a sibling or romantic partner), and items from the sleep routine/consistency (bedtime routine, trying to fall asleep every night at the same time, waking up every morning at the same time) and bedtime technology use (watching television, gaming, phone/computer use) domains. The most recently validated version of the PSPQ also includes items on sleep environment. However, these were not available at the time of the Dutch translation and were therefore not included in the study. Each item was rated on a 5-point Likert scale from 1 = never to 5 = always, in the last seven days, and added individually to the analyses. We additionally assessed whether participants were sleeping accompanied (either with someone in one room or in one bed) or alone.

### 2.4. Statistical Analyses

For all analyses, IBM SPSS version 26 was used. The prevalence rates of four groups were calculated, for patients with: (1) both insomnia and daytime fatigue, (2) insomnia only (3) daytime fatigue only, or (4) no insomnia or daytime fatigue symptoms. Furthermore, the proportion of patients in each clinical severity category of insomnia (based on the ISI score) was calculated for the total group and per insomnia subgroup. Multinomial logistic regression models were applied to determine risk factors for belonging to any of the three insomnia and/or daytime fatigue groups compared to patients with no symptoms (reference group; categorical dependent variable). The following risk factors (independent variables) were evaluated: (1) sociodemographic—sex, age group, educational level, living situation; (2) medical—age at diagnosis, cancer diagnosis, type of oncologic treatment, time since end of treatment, co-morbid health condition, and (3) sleep behaviors (scores; see paragraph above). Univariate analyses were computed for each risk factor and those with a *p*-value < 0.15 were added to the multivariable model, which entailed: sex, age group, educational level, co-morbid health condition, cancer diagnosis, age at diagnosis, time since end of treatment, sleeping accompanied, bedtime technology use—gaming and mobile phone/computer use, bedtime routine, needing someone else to fall asleep, bedtime consistency, and wake time consistency. Hereafter, a stepwise backwards selection method was applied in which variables with the highest *p*-values were deleted, until only factors with a significance level of a *p*-value < 0.05 were kept in the final model [46]. Missing data on the outcomes (ISI or daytime fatigue) were below 2% and were therefore handled by listwise deletion.

## 3. Results

### 3.1. Sample Characteristics

**Figure 1:**
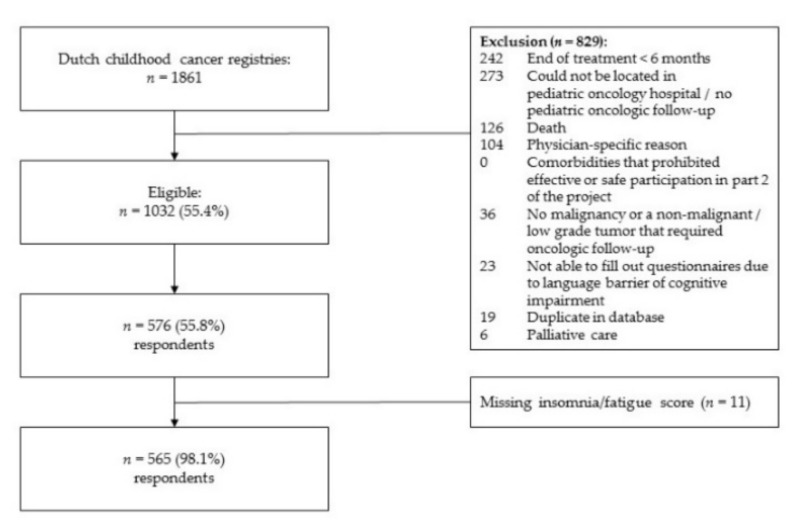
Flowchart of study inclusion.

We invited 1032 patients, of whom 576 participated (response rate 55.8%). Responders did not differ from non-responders in age, age at diagnosis, diagnosis type, and time since diagnosis (*p* > 0.05). However, females responded more frequently (*χ*2 = 8.18, *p* < 0.01). Furthermore, 11 patients had missing scores in either the insomnia or daytime fatigue items, so therefore the total sample used in the analyses was 565; see Figure 1. See Table 1 for a description of the total sample on key characteristics and per subgroup of insomnia–daytime fatigue, insomnia only, daytime fatigue only and no symptoms.

### 3.2. Prevalence Rates of Insomnia and Daytime Fatigue

**Figure 2:**
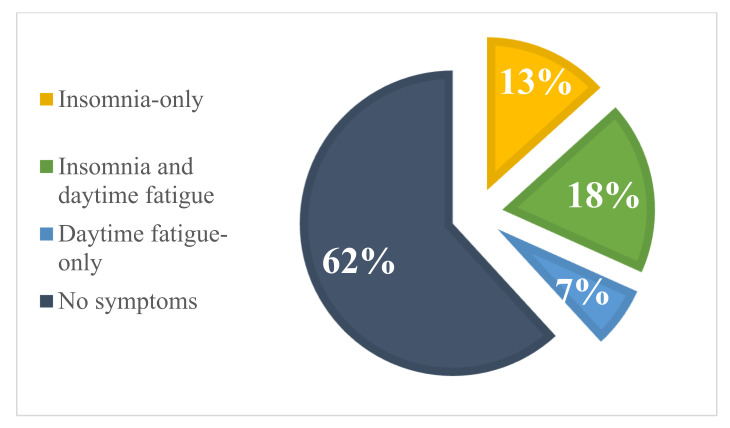
Prevalence of subgroups in all patients (*n* = 565).

The majority of patients did not report insomnia or daytime fatigue (61.8%, *n* = 349), followed by those who reported both insomnia and daytime fatigue (*n* = 104; 18.4%), insomnia only (*n* = 75; 13.3%) or daytime fatigue only (*n* = 37; 6.5%); see Figure 2.

Insomnia was reported by 31.7% of patients (*n* = 179) and daytime fatigue by 25.0% of patients (*n* = 141). Of those who reported insomnia and/or daytime fatigue symptoms (*n* = 216), 48% reported both symptoms (*n* = 104), 34.7% insomnia only (*n* = 75), and 17.1% daytime fatigue only (*n* = 37); see Figure S1. Of those with daytime fatigue, the majority reported insomnia (73.8%, *n* = 104/141). Moreover, in those with insomnia, more than half reported daytime fatigue (58.1%, *n* = 104/179).

### 3.3. Clinical Severity Ranges of Insomnia in the Total and Subgroups

The majority of patients with insomnia had subclinical symptoms (20.0%), followed by moderate severity (9.7%) and severe insomnia (1.9%); see Table 2. Insomnia severity was higher in the insomnia–daytime fatigue group compared to the insomnia-only subgroup (*t* (177) = −3.48, *p* < 0.001), with more moderate severity (45.1% versus 12.0%) and no severe insomnia in the insomnia-only group versus 7.7% in the insomnia–daytime fatigue group.

### 3.4. Risk Factors of Insomnia and Daytime Fatigue Groups

Significant risk factors in the multivariable final model are depicted in Table 3. Females and those who had a co-morbid health condition were at risk for symptoms in all groups compared to the no-symptom group. Young adults (vs. adolescents) were at risk in the insomnia–daytime fatigue group and daytime fatigue-only group, but not significantly in the insomnia-only group. Less time since end of treatment was a significant risk factor in the insomnia-only group. In both insomnia groups, needing someone else to fall asleep and inconsistent wake-up times were significant risk factors. Trying to fall asleep every night at the same time was significant in the insomnia–daytime fatigue group and gaming around bedtime was a significant risk factor in the insomnia-only group.

In the univariate analyses, there were no significant associations with living situation, type of oncologic treatment, and watching television around bedtime (*p* > 0.05); see Supplementary Table S2. There was a significant association with older age at diagnosis in the insomnia–daytime fatigue group (OR 1.12, 95%CI 1.03–1.20, *p* < 0.05) and the daytime fatigue-only group (OR 1.18, 95%CI 1.03–1.34, *p* < 0.05). Furthermore, there was a higher risk of insomnia only in the hemato-oncology diagnosis group (OR 2.14, 95%CI 1.01–4.51, *p* < 0.05). Another significant association was shown for sleeping accompanied (OR 3.34, 95%CI 1.64–6.82, *p* < 0.01) and a more consistent bedtime routine (OR 1.25, 95%CI 1.06–1.47, *p* < 0.01) in the insomnia–daytime fatigue group and more use of mobile phones/computers around bedtime in the insomnia–daytime fatigue (OR 1.20, 95%CI 1.00–1.44, *p* < 0.05) and daytime fatigue-only groups (OR 1.45, 95%CI 1.05–2.00, *p* < 0.05).

## 4. Discussion

In this cross-sectional national cohort study in 565 adolescent and young adult childhood cancer patients in follow-up after treatment, we found that the majority of patients did not have insomnia and daytime fatigue symptoms (61.8%). However, a large proportion did suffer from insomnia symptoms (31.7%) and daytime fatigue (25.0%). In those who had symptoms, we could distinguish three subgroups—insomnia–daytime fatigue (48.1%), insomnia only (34.7%), and daytime fatigue only (17.1%)—showing that there is a common co-occurrence of both symptoms. Those in the insomnia–daytime fatigue group suffered from more severe insomnia symptoms compared to the insomnia-only group. We could determine several risk factors for the insomnia–daytime fatigue subgroups, which were mostly sociodemographic and sleep behavioral factors. Most cancer-related factors were not associated with insomnia or fatigue.

Overall, this study adds knowledge on the complex relationships between insomnia symptoms and daytime fatigue. We found that the co-occurrence of insomnia symptoms and daytime fatigue was common in adolescent and young adult pediatric oncology groups after treatment. In those with daytime fatigue, the majority also had insomnia symptoms, and in those with insomnia, more than half had daytime fatigue. Those with both insomnia symptoms and daytime fatigue also reported more severe insomnia symptoms than those with insomnia only, which is in line with the previous literature [47]. The subgroup with co-occurring insomnia–daytime fatigue might be a more vulnerable patient group for poorer health outcomes for various reasons: (1) co-morbid health problems were more common in this group, which is also in line with previous insomnia research [48]; (2) in the general population, having insomnia and fatigue was related to more impaired health-related quality of life than those with less symptoms [9], and (3) in a cohort of non-irradiated pediatric acute lymphoblastic leukemia survivors, the cluster with more severe fatigue and sleep problems had more neurocognitive deficiency than those with milder symptoms [28]. Furthermore, insomnia and daytime fatigue might overall also cluster with several psychological complaints that have been reported by survivors, such as depression and anxiety [49], which has also been found in the general population [50]. There is a complex interaction between the psychological, somatic, and social factors influencing insomnia and fatigue [14,15,16,17,18]. However, our results mainly focused on the bidirectional relationship between fatigue and insomnia, and are limited to cross-sectional conclusions. In future research, the onset of insomnia symptoms and daytime fatigue, in relation to other important factors within the complex interaction between symptoms, in a longitudinal design, might offer insight into whether insomnia perpetuates fatigue, or vice versa.

The prevalence of overall daytime fatigue (25.0%) in our study was comparable to what was previously reported by children with chronic diseases (21.2%), including childhood cancer patients after treatment, and increased compared to the general population [14]. It is also in line with rates reported in other Dutch childhood cancer survivor cohorts of 20.8–31.3%, which were significantly higher than reported in siblings, in which 13.5% reported fatigue [51]. Moreover, the insomnia rate of approximately one third of patients reporting symptoms is similar to previous studies in childhood cancer survivors, reporting 31.0% [1] and 30.8%, compared to 25.7% in siblings [2], and comparable to what has been found in adult cancer survivors of 23–44% 2 to 5 years after treatment [16]. In the general population, insomnia symptom rates vary between 10 and 28% depending on the population, definition, and measurement methodology used [52]. The majority of our population reported subthreshold insomnia symptoms. Importantly, not only clinical symptoms, but also subthreshold insomnia symptoms, have a relevant, negative effect on patients’ well-being, and are related to a poorer quality of life [53,54]. Although most treatment research is based on clinical insomnia, one recent study indicated that subthreshold insomnia can also be effectively treated [55].

Previous studies have shown that patients have a need for support for their sleep and fatigue symptoms after active treatment has ended [4,56,57]. However, they often experience barriers with reporting sleep problems to their healthcare professional [58]. Therefore, active assessment—for example, by using patient-reported outcome measures (PROMs)—might help to recognize symptoms and incorporate fatigue–insomnia management within routine clinical care [59,60]. For both symptoms, effective treatments are available. For insomnia, cognitive behavior therapy for insomnia (CBT-i) is the first choice of treatment [34] in the general population. In the current clinical guidelines for fatigue, physical exercise training, mindfulness, or cognitive behavioral therapy for fatigue are recommended [11,33]. In (adult) cancer survivors, CBT programs for insomnia and fatigue have been shown to be effective in improving functional health outcomes in a recent meta-analysis [61]. Considering the co-occurrence of insomnia and fatigue, adding CBT-i elements to fatigue management strategies, such as sleep hygiene, sleep routines, sleep restriction, and addressing cognitions about sleep, might be useful in subgroups of fatigued patients. However, previous research has found that CBT-i is not as effective for the treatment of daytime symptoms [62], so adding fatigue management to insomnia treatment can also potentially optimize treatment outcomes. Future research should assess the added value of customizing insomnia and fatigue treatments to address both symptoms and incorporating insomnia and fatigue routine assessment within clinical care.

Risk factors to distinguish the three symptom groups might contribute to the better recognition of patients at risk and to customized symptom management strategies. Some factors were important in all groups, such as being female and having a co-morbid health condition, which are known risk factors for both insomnia and fatigue [51,63,64]. An explanation might be that females are also more vulnerable to other mental health complaints, such as anxiety or depression, which often co-occur with insomnia symptoms and daytime fatigue [65,66]. Furthermore, women with insomnia are also more likely to report daytime consequences, such as fatigue. In adolescents, these sex differences tend to emerge in line with pubertal maturation after first menses have started [67]. The co-morbid health conditions in this study were diverse and included both medical and psychological conditions/symptoms (see Table S1). They were also self-reported, which might have led to some reporting bias, and we could not distinguish between cancer-related effects and unrelated co-morbidities. However, considering the risk of late effects in childhood cancer survivors [68], this finding remains relevant. Therefore, clinicians and researchers should be aware of insomnia and daytime fatigue symptoms in patients with co-morbidities.

Apart from less time after treatment in the insomnia-only group, most cancer-related factors (type of diagnosis, type of oncology treatment, or age at diagnosis) did not have differentiating effects for insomnia or fatigue. We expected that within the cancer diagnosis groups, the central nervous system tumors were a risk factor, since some previous studies reported this for fatigue [51] and sleep [69,70], although results have been conflicting [2,11]. Presumably, there is a subgroup of CNS tumors at higher risk for sleep problems, such as tumors within the suprasellar region that interfere with sleep–wake regulation [71,72]. However, as these tumors are rare, they only represented a small group in this study, which might explain why no differences in diagnosis type were found with regard to CNS tumors. An explanation as to why insomnia-only patients that were closer to the end of treatment were more at risk might be represented by the residual effects of the treatment period. For example, fatigue/sleep may be disrupted in acute lymphoblastic leukemia due to corticosteroid treatment-related side effects [20,21,73]. Rates of insomnia during treatment tend to be higher (up to 75% [74]) than those in survivors of around one third [1,2,3]. A similar pattern has been found in parents of children with ALL [75]. Those closer to the end of treatment and younger (adolescent) patients might also suffer from more habitual insomnia that is influenced by sleep hygiene [10]. This was also shown in our results, since gaming before bedtime and inconsistent wake-up times were risk factors in this group.

Other sleep behavioral factors that were also significant in our multivariable model were: needing someone else to fall asleep (either a parent or significant other, such as a sibling or romantic partner) and bedtime and wake time consistency. Needing someone else to fall asleep in both insomnia groups was remarkable, considering that our population was 12–26 years old and on average three years after treatment. This was previously described in younger pediatric cancer patients (aged 5–17 years old) during active cancer treatment [22]. Our findings suggest that this sleep behavior is maintained for a longer time after treatment, and is associated with having insomnia. Patients with insomnia more often indicated trying to fall asleep at the same time each night, suggesting in the wording of the item a behavioral component of a struggle of falling asleep. A hypothesis might be that people with insomnia often receive the advice to sleep at consistent times and since some research shows that this group may have the habit of pushing themselves to succeed instead of changing tactics [76]. Therefore, these behaviors might result in staying awake due to increased arousal when consistent bedtime behavior is not helpful enough to change the sleeping pattern and relieve the insomnia complaints. Inconsistent wake times in the adolescent and young adult age groups as a risk factor is in line with the literature about the misalignment of the biological clock of this developmental age group with societal needs, such as school times [77]. Since sleep behaviors are modifiable factors, including them in sleep health prevention material after the end of cancer treatment and in interventions is important to consider. There is increased awareness for incorporating preventative healthy lifestyle interventions for adolescent and young adult childhood cancer survivors due to their health vulnerabilities [78,79,80]. Addressing insomnia/sleep and fatigue within these preventative healthy lifestyle programs might be a valuable addition.

### Strengths and Limitations

The primary strength of this study is that it consists of a large heterogeneous national cohort representing all types of childhood cancer diagnoses. However, the conclusions are also restricted by several limitations. First, we measured daytime fatigue using a single item that has not been validated. Some studies suggest that one item measuring daytime fatigue is sufficient when compared to a full-scale questionnaire [81,82], but some also report that it is not accurate enough [83]. However, the current prevalence rate of 25.1% is comparable to the prevalence of 21.2% for severe fatigue found in children with chronic diseases including childhood cancer post-treatment using a validated questionnaire [14]. Second, the recall period of the ISI was seven days, compared to three months of the daytime fatigue item. The daytime fatigue group might therefore represent more severe complaints persisting for a longer period of time compared to the insomnia group. Third, we could not compare our results to normative data from a representative Dutch adolescent/young adult sample. Finally, we did not measure other potential but important risk factors for insomnia/daytime fatigue symptoms, such as psychological problems with which they may cluster, such as depression or anxiety, but also factors such as non-employment, exercise, smoking, higher body mass index (BMI) [11,51], or family relational factors, such as parental sleep problems and parenting problems [84,85,86].

## 5. Conclusions

Insomnia symptoms and daytime fatigue are common and often co-occur in pediatric oncology groups after treatment. While current fatigue guidelines do not include insomnia symptoms, healthcare providers should inquire about insomnia as this potentially provides additional options to improve patients’ quality of life. Future research should evaluate the effectiveness of adding insomnia monitoring and treatment to fatigue management and explore potential prevention strategies after the end of treatment.

## Figures and Tables

**Table 1:** Sample characteristics per total group and per insomnia–daytime fatigue subgroup.

	Total Group (*n* = 565)	Insomnia-Daytime Fatigue Subgroups			
		Insomnia and Daytime Fatigue (*n* = 104)	Insomnia Only (*n* = 75)	Daytime Fatigue Only (*n* = 37)	No Symptoms (*n* = 349)
Age at study invitation (y), mean (SD) Age group, % Adolescents (12–17y) Young adult (18–26y)	17.0 (2.9) 57.3 42.8	17.8 (2.7) 40.4 59.6	16.6 (2.0) 68.0 32.0	18.5 (2.7) 35.1 64.9	16.7 (2.9) 62.2 37.8
Sex, % Female	49.6	66.3	57.3	78.4	39.8
Current educational level, % Low Middle High	23.9 56.6 18.2	27.9 51.9 17.3	25.3 64.0 10.7	24.3 40.5 32.4	22.3 58.2 18.6
Country of birth, % The Netherlands Other	95.2 3.4	90.4 7.7	94.7 5.3	94.6 2.7	96.8 1.7
Age at diagnosis (y), mean (SD)	12.8 (3.2)	13.5 (3.3)	13.0 (2.9)	13.9 (2.6)	12.5 (3.2)
Time since diagnosis in years, mean (SD)	4.0 (2.4)	4.1 (2.4)	3.5 (1.9)	4.3 (2.5)	4.0 (2.4)
Diagnosis groups, % Hemato-oncology Neuro-oncology Solid	44.1 23.7 32.2	45.2 28.8 26.0	49.3 13.3 37.3	43.2 21.6 35.1	42.7 24.6 32.7
Time since end of treatment in years, mean (SD)	3.2 (2.2)	3.3 (2.1)	2.7 (1.8)	3.2 (2.4)	3.3 (2.3)
Type of oncologic treatment, % No treatment Chemotherapy Radiation Surgery SCT Mixed/Other	1.4 73.1 25.5 54.7 6.2 4.8	0.9 67.3 24.0 63.5 9.6 6.7	- 73.3 21.3 54.7 6.7 6.7	2.7 73.0 24.3 54.1 5.4 -	2.0 74.8 26.9 52.1 5.2 4.0
Comorbid health problems, % Yes	17.9	31.7	22.7	32.4	11.2
Sleeping accompanied, % Yes	7.4	15.4	6.7	8.3	5.2
Sleep behaviors, mean (SD) Needing someone else to fall asleep Bedtime routine Trying to fall asleep every night at the same time Waking up every morning at the same time Bedtime technology TV Games Phone/computer	1.18 (.67) 3.43 (1.44) 3.35 (1.20) 3.43 (1.12) 3.60 (1.20) 2.10 (1.28) 3.83 (1.31)	1.52 (1.08) 3.75 (1.15) 3.64 (1.04) 3.13 (1.14) 3.66 (1.03) 2.07 (1.22) 4.04 (1.07)	1.25 (.81) 3.43 (1.31) 3.43 (1.27) 3.33 (1.08) 3.65 (1.07) 2.25 (1.35) 3.69 (1.38)	1.05 (.23) 3.59 (1.46) 3.46 (1.10) 3.24 (1.09) 3.46 (1.32) 1.68 (1.18) 4.27 (1.05)	1.08 (.44) 3.32 (1.53) 3.24 (1.23) 3.57 (1.11) 3.58 (1.26) 2.13 (1.29) 3.74 (1.36)

Notes. SCT = stem cell transplantation, y = years, SD = standard deviation.

**Table 2:** Insomnia clinical severity categories and ISI scores per total and subgroup.

	Total Group (*n* = 565)	Insomnia-Daytime Fatigue Subgroups			
		Insomnia and Daytime Fatigue (*n* = 104)	Insomnia Only (*n* = 75)	Daytime Fatigue Only (*n* = 37)	No Symptoms (*n* = 349)
Insomnia categories (*n*, %): No clinically significant insomnia (ISI: 0–7) Subthreshold insomnia (ISI: 8–14) Clinical insomnia (moderate severity, ISI: 15–21) Clinical insomnia (severe, ISI: 22–28)	386 (68.3) 115 (20.4) 56 (9.9) 8 (1.4)	- 49 (47.1) 47 (45.1) 8 (7.7)	- 66 (88.0) 9 (12.0) -	37 (100.0) - - -	349 (100.0) - - -
ISI score, mean (SD)	6.15 (5.84)	14.89 (4.72)	11.41 (2.96)	4.59 (1.69)	2.58 (2.18)

**Table 3:** Risk factors for insomnia–daytime fatigue subgroups compared to no symptoms: results of the final multinomial regression model, multivariable (OR and 95% CI).

Reference: No Symptoms (*n* = 349)	Insomnia and Daytime Fatigue (*n* = 104)	Insomnia Only (*n* = 75)	Daytime Fatigue Only (*n* = 37)
Female	3.08 (1.75–5.41) ***	2.41 (1.34–4.35) **	6.38 (2.44–16.70) ***
Age group: young adults (ref: adolescents)	3.11 (1.75–5.54) ***	1.09 (0.55–2.14)	3.95 (1.58–9.90) **
High educational level (ref: low/middle)	0.36 (0.17–0.76) **	0.58 (0.22–1.50)	0.95 (0.37–2.47)
Having a comorbid health condition	4.53 (2.47–8.31) ***	3.00 (1.50–5.98) **	4.32 (1.79–10.44) **
Time since end of treatment (in years)	0.93 (0.83–1.05)	0.86 (0.75–0.99) *	0.85 (0.70–1.02)
Needing someone else to fall asleep	1.79 (1.25–2.56) **	1.56 (1.04–2.33) *	0.63 (0.20–1.97)
Trying to fall asleep every night at the same time	1.40 (1.10–1.77)**	1.19 (0.93–1.51)	1.10 (0.77–1.56)
Waking up every morning at the same time	0.65 (0.51–0.82) ***	0.77 (0.60–1.00) *	0.72 (0.51–1.03)
Bedtime technology use-gaming	1.17 (0.94–1.45)	1.27 (1.03–1.59) *	0.98 (0.67–1.44)

Note. **Bold** odds ratios were significant, if significant asterisks were added * < 0.05, ** < 0.01, *** < 0.001.

## Data Availability

The data presented in this study are available on request from the corresponding author. The data are not publicly available due to privacy restrictions.

## Supplementary Materials

The following supporting information can be downloaded at: https://www.mdpi.com/article/10.3390/cancers14143316/s1, Table S1: Overview of self-reported co-morbid health conditions; Table S2: Univariate analyses of determinants of insomnia–daytime fatigue subgroups within multinomial regression analyses (OR and 95% CI); Figure S1: Overlap between insomnia and daytime fatigue in the total group of patients with either insomnia and/or daytime fatigue symptoms (*n* = 216).
