# Supplementary material for: Insomnia Symptoms and Daytime Fatigue Co-Occurrence in Adolescent and Young Adult Childhood Cancer Patients in Follow-Up after Treatment: Prevalence and Associated Risk Factors
Source: Cancers (Basel). 2022 Jul 7;14(14):3316. doi: 10.3390/cancers14143316 (PMC9313407; doi:10.3390/cancers14143316)
Supplement: Supplementary file 1 [file cancers-14-03316-s001.zip › cancers-1772707-supplementary.pdf]

cancers

Supplementary material.

Insomnia symptoms and daytime fatigue co–occurrence in adolescent and young adult childhood cancer patients in follow–up after treatment: prevalence and associated risk factors

Shosha H. M. Peersmann[1,2], Martha A. Grootenhuis[17], Annemieke van Straten3, Wim J.E. Tissing[1,4], Floor Abbink[2], Andrica C.H. de Vries[1,5], Jacqueline Loonen[6], Helena J. H. van der Pal[1], Gertjan J.L. Kaspers[1,2] and Raphaële R. L. van Litsenburg[1,2*]

Table S1. Overview of self–reported co–morbid health conditions (n = 101)*.

Type of condition	Number of cases
Psychological	20
Neurological	15
Respiratory	11
Behavior and developmental	8
Gastro–intestinal	8
Endocrinological	8
Allergies	5
Orthopedic	5
Nephrological	4
Auto–immune disorders	4
Congenital and genetic abnormalities	3
Cardiovascular	2
Other	14

*Note. Some patients reported multiple types of conditions, the total number of cases does therefore not count up to the total n = 101.

Table S2. Univariate analyses of determinants of insomnia–fatigue subgroups within multinomial regression analyses (OR and 95% CI).

Reference:<br>No symptoms (*n* = 349)	Insomnia and Fatigue<br>(*n* = 104)	Insomnia–only (*n* = 75)	Fatigue–only (*n* = 37)
Female	**2.98 (1.23–3.37)*****	**2.03 (1.23–3.37)****	**6.26 (2.67–14.68)*****
Young adults (ref: adolescents)	**2.43 (1.55–3.80)*****	0.77 (.46–1.32)	**3.04 (1.49–6.17)****
High educational level (ref: low/middle)	0.93 (.52–1.65)	0.52 (.24–1.13)	**2.17 (1.03–4.56)***
Living with parents	0.74 (.36–1.49)	1.13 (.45–2.81)	0.49 (.19–1.27)
Having a comorbid health condition	**3.74 (2.20–6.37)*****	**2.46 (1.30–4.66)****	**3.91 (1.81–8.43)****
Age at diagnosis in years	**1.12 (1.03–1.20)****	1.05 (.97–1.14)	**1.18 (1.03–1.34)***
Cancer diagnosis:<br>Hemato<br>Solid<br>(ref: CNS)	0.90 (.53–1.54)<br>0.68 (.38–1.22)	**2.14 (1.01–4.51)***<br>2.11 (.97–4.58)	1.15 (.47–2.81)<br>1.23 (.49–3.09)
Type of oncologic treatment[1]:<br>Surgery only<br>Chemotherapy with or without surgery<br>(ref: Radiation and/or SCT)	1.17 (.63–2.15)<br>0.99 (.60–1.64)	1.09 (.53–2.24)<br>1.10 (.62–1.95)	0.77 (.28–2.13)<br>0.95 (.45–2.01)
Time since end of treatment (in years)	1.00 (.90–1.10)	**0.86 (.76–.98)***	0.98 (.83–1.14)
Sleeping together	**3.34 (1.64–6.82)****	1.31 (.47–3.66)	1.67 (.47–5.97)
Needing someone else to fall asleep	**2.31 (1.64–3.27)*****	**1.74 (1.15–2.62)****	0.82 (.27–2.47)
Bedtime routine	**1.25 (1.06–1.47)****	1.05 (.88–1.25)	1.14 (.90–1.46)
Trying to fall asleep every night at the same time	**1.35 (1.11–1.64)****	1.14 (.92–1.41)	1.17 (.87–1.56)
Waking up every morning at the same time	**0.71 (.58–.86)*****	0.82 (.66–1.03)	0.77 (.57–1.04)
Bedtime technology:<br>TV<br>Gaming<br>Phone/computer	1.06 (.88–1.27)<br>0.96 (.81–1.15)<br>**1.20 (1.00–1.44)***	1.05 (.85–1.30)<br>1.08 (.89–1.30)<br>0.97 (.81–1.17)	0.92 (.70–1.21)<br>**0.72 (.52–.99)***<br>**1.45 (1.05–2.00)***

Note. if significant asterisks were added * <.05, **<.01 ***<.001.

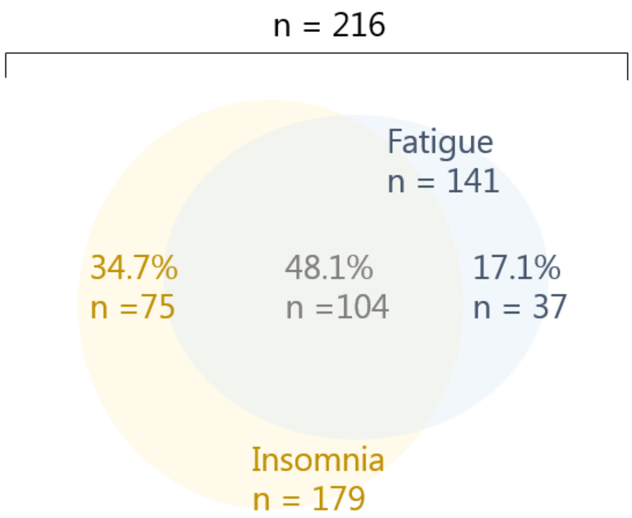

Figure S1. Overlap between insomnia and fatigue in the total group of patients with either in– somnia and/or fatigue symptoms (n = 216).
